# An α-1,6-and α-1,3-linked glucan produced by *Leuconostoc citreum* ABK-1 alternansucrase with nanoparticle and film-forming properties

**DOI:** 10.1038/s41598-018-26721-w

**Published:** 2018-05-29

**Authors:** Karan Wangpaiboon, Panuwat Padungros, Santhana Nakapong, Thanapon Charoenwongpaiboon, Martin Rejzek, Robert A. Field, Rath Pichyangkura

**Affiliations:** 1 Department of Biochemistry, Faculty of Science, Chulalongkorn University, Bangkok, 10330 Thailand; 2 Department of Chemistry, Faculty of Science, Chulalongkorn University, Bangkok, 10330 Thailand; 3 Department of Chemistry, Science, Ramkhamhaeng University, Bangkok, 10240 Thailand; 4 Department of Biological Chemistry, John Innes Centre, Norwich Research Park, Norwich, NR4 7UH UK

## Abstract

Alternansucrase catalyses the sequential transfer of glucose residues from sucrose onto another sucrose molecule to form a long chain polymer, known as “alternan”. The alternansucrase-encoding gene from *Leuconostoc citreum* ABK-1 (*Lcalt*) was successfully cloned and expressed in *Escherichia coli*. *Lcalt* encoded *Lc*ALT of 2,057 amino acid residues; the enzyme possessed an optimum temperature and pH of 40 °C and 5.0, respectively, and its’ activity was stimulated up to 2.4-fold by the presence of Mn^2+^. Kinetic studies of *Lc*ALT showed a high transglycosylation activity, with K_m_ 32.2 ± 3.2 mM and kcat 290 ± 12 s^−1^. Alternan generated by *Lc*ALT (*Lc*-alternan) harbours partially alternating α-1,6 and α- 1,3 glycosidic linkages confirmed by NMR spectroscopy, methylation analysis, and partial hydrolysis of *Lc*-alternan products. In contrast to previously reported alternans, *Lc*-alternan can undergo self-assembly, forming nanoparticles with an average size of 90 nm in solution. At concentrations above 15% (w/v), *Lc*-alternan nanoparticles disassemble and form a high viscosity solution, while this polymer forms a transparent film once dried.

## Introduction

Carbohydrate polymers play many important roles in nature, as the main energy material, as key structural components, and in biological recognition processes. They are widely spread in all organisms, from plants and animals to bacteria, fungi and protozoa^1,2^. Exopolysaccharides (EPSs) from microorganisms are important carbohydrates in the field of biotechnology^3^. They are used in foods as sweeteners, prebiotics and food extenders. Moreover, EPSs have been shown to have potential medical applications, as adjuvants and blood plasma substitutes, and they are also used as matrices in column chromatography^4,5^.

EPSs can be produced by glucansucrases, most of which belong to the CAZy glycoside hydrolase family 70 (GH70)^4,6,7^, these enzymes transfer glucose residues from a sucrose donor to produce a variety of isomeric glucan polymers. Currently, four main types of GH70 glucansucrases have been reported: mutansucrase^8^, dextransucrase^9^, reuteransucrase^10^ and alternansucras^11^. They are distinguished from each other by the type of glycosidic linkages found in their glucan products. Numerous glucansucrases have been reported in the past fifty years, with diversities in the enzyme structure, property, source and product^12–15^. For example, reuteransucrase from *Lactobacillus reuteri* ATCC 55730^16^ obviously showed higher hydrolytic rate than that of *Lactobacillus reuteri* 121^10^. Dextransucrase from different isolates of *Leuconostoc citreum* and *Leuconostoc mesenteroides* were reported to have their own unique degree and type of branching^17–19^.

Alternansucrase (ALT, EC 2.4.1.140) produces an insoluble polymer called “alternan”, which has alternating α-1,6 and α-1,3 glycosidic linkages as judged by using ^13^C NMR spectroscopy and methylation analysis^20^, partially hydrolysed product analysis, and enzymatic analysis^11^. Alternansucrase from *Leuconostoc mesenteroides* NRRL B-1355 (*Lmalt*) has been successfully cloned and partially characterised^21^, although the enzyme was unstable and rapidly degraded. Deletion of the *Lm*ALT carboxy-terminus increased its stability without effecting the linkage pattern of its’ product, as confirmed by ^13^C NMR spectroscopy^22^. The genomic sequence of *Leuconostoc citreum* KM20 revealed a cluster of three putative dextransucrases and an alternansucrase gene^23^. However, there are no reports on the cloning and characterisation of *alt* from *L*. *citreum*. Nevertheless, Musa *et al*. (2014) have produced and partially purified ALT from *Leuconostoc citreum* SK24 cell culture, and the enzyme was used for the biotransformation of stevioside^24^.

In this report, we describe the cloning, expression and biochemical characterisation of alternansucrase from *Leuconostoc citreum* ABK-1 (*Lc*ALT), as well as the characterisation of its alternan product. Furthermore, the properties of the alternan product, such as polymer solubility, nanoparticle and film-forming properties, were also explored.

## Materials and Methods

### Culturing of *L*. *citreum* ABK-1

*L*. *citreum* ABK-1 was isolated from Khow-tom-mud (Thai dessert) and its identity was subsequently confirmed by 16 s rRNA sequencing. It was cultured on LB agar plate containing 5% (w/v) sucrose or 1% (w/v) glucose at 30 °C or in LB broth containing 5% (w/v) sucrose or 1% (w/v) glucose at 30 °C with rotary shaking at 250 rpm for overnight.

### Cloning of *Lcalt* gene

Genomic DNA of *L*. *citreum* ABK-1 was extracted and the *alt* gene was amplified using PrimeSTAR^®^ DNA polymerase (TAKARA). PCR conditions were as follows: initial denaturation at 98 °C for 30 sec, 35 cycles of 98 °C for 10 sec, 57 °C for 10 sec and 72 °C for 5.30 min, and final extension at 72 °C for 10 min, using forward primer (F_NcoAlt: 5′ GGGAGAGTAATCCATGGAACAACAAGAAAC 3′) and reverse primer (R_XhoAlt: 5′ CCGGAGCTCTTAAGCTTGCAAAGCACGCTTATC 3′). The primer set was designed based on the GenBank-deposited genome sequence of *L*. *citreum* KM20 [NC_010471]. *Nco*I and *Xho*I restriction sites (underlined) were incorporated in the forward and reverse primers, respectively. The PCR product was directionally cloned into pET19b (Novagen) and transformed into *Escherichia coli* DH5α. Colonies were randomly picked and the gene insertion was initially analysed by restriction enzyme digestion (NEB). The positive clones were selected and further confirmed by nucleotide sequencing (1^st^ BASE). All molecular techniques were performed according to literature procedures^25^.

### Expression of *Lcalt* gene in *E*. *coli* and production of recombinant *Lc*ALT

The recombinant plasmid (pETalt) was transformed into *E*.*coli* BL21 (DE3). The transformants were grown in LB broth containing appropriate antibiotic at 37 °C with rotary shaking at 250 rpm until OD_600_ reached 0.6. The cell cultures were then induced with various concentrations of isopropyl β-D-1-thiogalactopyranoside (IPTG), from 0.2 - 1 mM, and the culturing temperature was shifted to 16 °C, 30 °C or 37 °C for 20 hr. The culture medium and cells were separated by centrifuging at 8,000 × g for 10 min. The cell pellets were re-suspended in 0.3 mL of buffer per 1 mL of culture volume, containing 50 mM sodium citrate buffer pH 6.2, 75 mM of sodium chloride, and 0.1% (v/v) Triton X-100. The cells were lysed by sonication, and the cell lysate was fractionated by centrifuged at 10,000 × g, 4 °C for 10 min. The crude supernatant containing the enzyme was collected and stored at 4 °C for further analysis.

### Purification of *Lc*ALT

The crude *Lc*ALT was dialysed against 25 mM citrate buffer pH 6.2 and applied to a DEAE-Toyopearl-650 M (Tosoh Bioscience) column (22 × 90 mm) equilibrated with the same buffer (0.7 mL/min of flow rate). The fractions containing enzymatic activity were pooled and ammonium sulfate was added to achieve a final concentration of 400 mM. Subsequently, the protein solution was loaded onto a Phenyl-Toyopearl-650 M (Tosoh Bioscience) column (22 × 90 mm) equilibrated with 25 mM citrate buffer pH 6.2, containing 400 mM ammonium sulfate. The column was eluted with a 350–150 mM ammonium sulfate gradient with flow rate of 0.7 mL/min. Fractions exhibiting enzymatic activity were pooled, dialysed, and analysed on 8% SDS-PAGE gel.

### *Lc*ALT enzymatic activity assay

Enzymatic reactions containing 2% (w/v) sucrose and 50 mM citrate buffer pH 5.0 in 0.5 mL total volume were incubated at 40 °C for 20 min. The reaction was stopped by adding 0.5 mL of 3,5-dinitrosalicylic acid (DNS) then boiled for 10 min. The total reducing sugar was determined by the DNS method, measuring the OD at 540 nm^26^. One unit of *Lc*ALT activity was defined as the amount of the enzyme that released 1 µmole of fructose per minute, using fructose as a standard.

### Biochemical characterisation of *Lc*ALT

#### Effect of temperature and pH on *Lc*ALT activity

The activity of *Lc*ALT was assayed at various incubation temperatures between 20–60 °C in 50 mM citrate buffer pH 6.0. The effect of pH was analysed in 50 mM of citrate buffer pH 3.0–6.0 and 50 mM phosphate buffer pH 6.0–8.0 at 37 °C. A 100 mU of purified enzyme (~2 µg) was used for these analyses. Relative activity at different temperatures and pHs were determined by the DNS method.

### Effect of metal ions and detergent on *Lc*ALT activity

The activity of alternansucrase was determined in 50 mM acetate buffer pH 5.0 at 40 °C in the presence of 10 mM CaCl_2_, CoCl_2_, CuCl_2_, FeCl_3_, MgCl_2_, MnCl_2_, ZnCl_2_, EDTA or 0.1% (v/v) Triton-X 100 using 100 mU of purified enzyme (~2 µg). Relative activity of *Lc*Alt in the presence of different chemicals was determined by the DNS method.

### Kinetic analysis of *Lc*ALT

Kinetic analysis was carried out in a 0.5 mL reaction volume using the following conditions: 0 to 100 mM sucrose, 50 mM sodium citrate buffer pH 5.0, 40 °C, and 100 mU of purified enzyme (~2 µg). The reaction was stopped by adding of 15 µL of 0.1 N NaOH. The quantity of reducing sugars was determined using the DNS assay and the glucose content arisen from sucrose hydrolysis was measured using a glucose oxidase assay (Glucose LiquiColor^®^, Human). Transglycosylation activity was calculated by subtracting the total reducing sugar with the amount of glucose, where the amount of glucose produced per minute indicates hydrolytic activity.

### Product analysis of *Lc*-alternan

#### NMR analysis of *Lc*-alternan

*Lc*-alternan was produced using 20% (w/v) sucrose with 5 U of *Lc*ALT per gram of sucrose in 50 mM sodium citrate buffer pH 5.0 at 37 °C in 100 mL total volume, overnight. The polymer was precipitated by addition of 1:1 volume of acetone then chilled on ice for 1 hr. The reaction was then centrifuged at 10,000 × g for 20 min. The *Lc*-alternan pellet was collected, re-suspended in deionised water, and dialysed against deionised water. The sample was lyophilised and stored for further experiments. *Lc-*alternan was dissolved in deionised water at 20% (w/v) and pre-treated to reduce the viscosity by ultrasonication (Sonics Vibra-Cell, at 50% power output) on ice, for 15 min. The sonicated *Lc-*alternan was lyophilised for further NMR analysis. ^13^C spectra of sonicated polymer were recorded at 100 MHz while ^1^H and COSY, HSQC and HMBC experiments were performed at 400 MHz in D_2_O or DMSO-d6 adding a few amount of trifluoroacetic acid (TFA).

### Methylation analysis

The samples were methylated with CH_3_I in a slurry of NaOH and DMSO. The methylation was performed twice. Then, it was completely hydrolysed using 2.5 M TFA for 4 hr at 100 °C. A myo-inositol standard was added and partially methylated monosaccharides were reduced with 0.5 M NaBD_4_ for 2.5 hr at room temperature. The samples were subsequently acetylated using excess acetic anhydride for 2.5 hr at 100 °C. Finally, the mixture of partially methylated alditol acetates was dissolved in dichloromethane and analysed by GC-MS^27^.

### Partial hydrolysis and analysis of *Lc*-alternan products by HPAEC-PAD

0.5 g of *Lc-*alternan in 20% (w/v) concentration was partially hydrolysed by heating at 100 °C for 12 min in 1 M HCl, then neutralised by adding an equivalent amount of NaOH. The partially hydrolysed alternan mixture was separated by size exclusion column chromatography, using a Biogel P-2 column (1000 × 22 mm) at 50 °C using flow rate of 0.5 mL/min. Compounds were monitored using an RI detector, and mass of oligosaccharide products was confirmed by MALDI-TOF mass spectrometry. The purified products were also analysed by HPAEC-PAD using a CarboPac^TM^ PA1 column (2 × 250 mm), in 150 mM NaOH and eluting with linear gradient of 0–150 mM sodium acetate in 150 mM NaOH with flow rate of 0.25 mL/min for 30 min. Finally, the sodium acetate concentration was raised up to 500 mM for 5 min. The pattern of oligosaccharides was compared with *Leuconostoc* spp. dextran hydrolysate (Sigma), isomaltose (Sigma), isomaltotriose (Sigma) nigerose (Sigma) and mutan hydrolysate produced from *Streptococcus sorbinus*^28^ (the mutansucrase (GTF-I) from *S*. *sorbinus* was obtained by expression of a synthetic gene (GenScript) in *E*. *coli* BL21 (DE3)).

### Determination of nanoparticle size distribution by Dynamic light scattering (DLS)

*Lc-*alternan was synthesised and then dialysed against deionised water without acetone precipitation. After that, it was analysed with a Zetasizer Nano ZS (Malvern) or lyophilised and then re-dispersed to 1% (w/v) in deionised water prior to analysis. Samples were pre-equilibrated for 1 min at 25 °C and analysed by using a refractive index value of 1.33, with 90° scattering optics at 633 nm.

### Solubility and film forming ability of *Lc-alternan*

Lyophilised *Lc-*alternan was re-suspended in deionised water, DMSO, 50% NaOH (w/v), at 1 and 20% (w/v) to investigate its’ solubility in different types of solvent. To study the effect of concentration on solubility, lyophilised *Lc-*alternan was re-suspended in deionised water at 1, 2.5, 5, 10, 15, 20 and 25% (w/v). To form films, 2% (w/v) *Lc-*alternan in deionised water was poured into a polypropylene box and air dried for overnight at room temperature.

### Transmission electron microscopy (TEM)

The lyophilised *Lc*-alternan was suspended in deionised water at 1% (w/v) and was diluted 100–10,000 folds. 1 µL of each concentration was dropped onto copper grids. The samples were dried in a desiccator for overnight, and TEM images were obtained using a Hitachi TEM H-7650.

## Results and Discussion

### Cloning and sequence analysis of *Lcalt*

**Figure 1:**
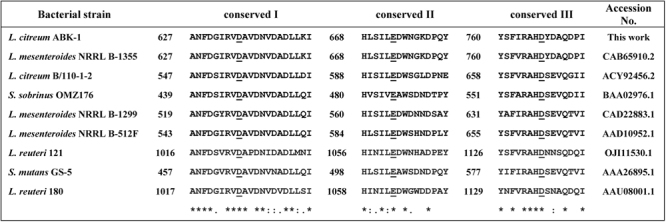
Alignment of are three conserved sequence motifs in the catalytic domain of glucansucrases. The sequences were compared by Clustal OMEGA. The symbol (^*^) represents identical residues, (:) shows highly conserved residues, and (.) represents conserved residues. Three catalytic residues were underlined.

The *alt* gene from *L*. *citreum* ABK-1 (*Lcalt*) [GenBank Accession number: KM083061.2], with an open reading frame of 6,174 bp, was successfully cloned into pET19b. The *Lcalt* gene encoded 2,057 amino acid residues with a calculated molecular weight of 228 kDa. The *Lcalt* nucleotide sequence showed 99% and 97% identity with *alt* of *L*. *citreum* KM20 [DQ489736.1] and *L*. *mesenteroides* NRRL-B1335 [AJ250173.2], respectively. The differences between *Lc*ALT and *Lm*ALT comprised 45 amino acid substitutions found in the enzyme, of which 32 substitutions were found in the catalytic domain (Supplemental Fig. S1). Only substitution of Val763 by Ile in *Lm*ALT was found in conserved domain III with no other changes in their conserved catalytic sequences (Fig. 1)^10,12^.

### Expression of *Lcalt* and purification of *Lc*ALT

**Figure 2:**
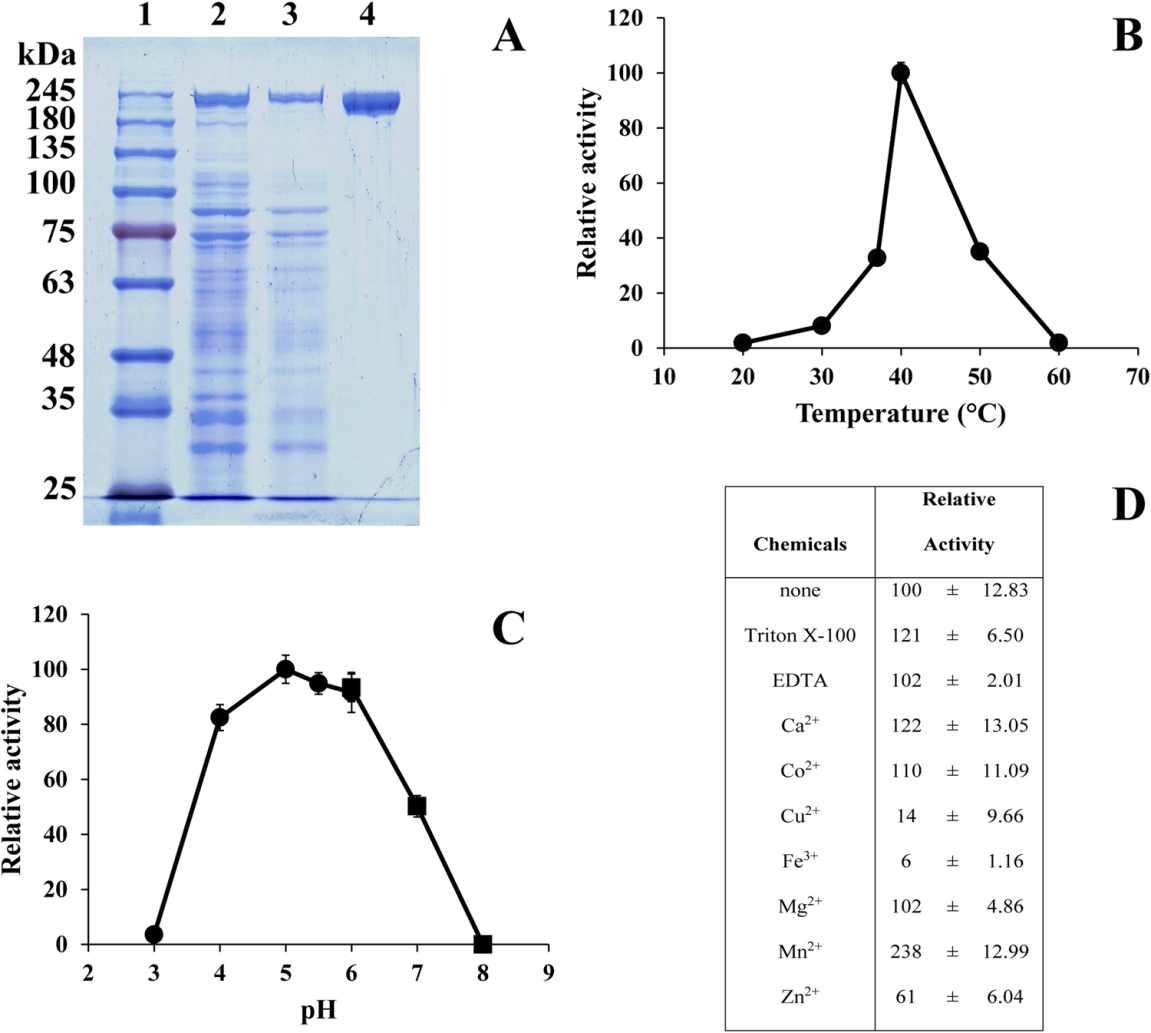
Characterisation of *Lc*ALT (**A**) SDS-PAGE analysis of purified *Lc*ALT. Lane 1: protein molecular weight marker, lane 2: crude enzyme (~8 µg protein), lane 3 and 4, purified proteins from DEAE-Toyopearl (~5 µg protein) and Phenyl-Toyopearl (~1 µg protein) columns, respectively. (**B**) Effect of temperature was assayed at 20–60 °C in 50 mM citrate buffer pH 5. (**C**) Effect of temperature was performed in (●) 50 mM of citrate pH 3.0–6.0 and in (■) 50 mM and phosphate buffer pH 6.0–8.0 at 37 °C. (**D**) Effect of metal ions and detergent was assayed in 10 mM various metal ions and EDTA while Triton X-100 was 0.1% (v/v).

The full length *Lcalt* gene was successfully expressed, and *Lc*ALT was produced as soluble intracellular protein in *E*. *coli* BL21 (DE3) (Fig. 2A). The optimum expression conditions were initial growth of cells at 37 °C temperature, then a shift of temperature to 16 °C after induction with 0.4 mM of IPTG. The highest yield of *Lc*ALT was achieved at 20 hr post induction. Production of native *Lc*ALT at low temperature reduced the expression level but promoted production of the soluble form of the protein. *Lc*ALT was stable since no degradation was observed in contrast to the previous report of *Lm*ALT^22^. Moreover, we found that the addition of 0.1% Triton X-100 (v/v) in the extraction buffer increased the solubility and yield of the enzyme. Crude *Lc*ALT was applied onto a DEAE column, the pooled fractions with enzymatic activity were then loaded onto a phenyl column. *Lc*ALT activity was eluted with ~260 mM ammonium sulphate. The purified *Lc*ALT had a specific activity of 34.7 U/mg protein and was recovered with approximate yield of 46%. The purified enzyme exhibited a high degree of apparent purity on SDS-PAGE, shown as a single band in Fig. 2A.

### Biochemical characterisation of *Lc*ALT

*Lc*ALT showed an optimal temperature of 40 °C (Fig. 2B), and an optimum pH of 5.0 (Fig. 2C), similar to *Lm*ALT where the optimal activity was reported at pH 5.5 and 40 °C. The activity of *Lc*ALT in the presence of selected compounds and metal ions was investigated, (Fig. 2D). In the presence of the non-ionic detergent, Triton-X 100, *Lc*ALT activity increased approximately 1.2-fold, possibly through reduced aggregation and increased solubility of the enzyme. Removal of divalent ions by the addition of EDTA had no effect on the enzymatic activity, unlike other glucansucrases, previously reported to be Ca^2+^ dependent enzymes^9,10,29^. However, addition of Ca^2+^ can slightly increase the activity of *Lc*ALT, 1.2-fold. Interestingly, the addition of 10 mM Mn^2+^ ion increased the activity of the enzyme over 2-fold (Fig. 2D).

The kinetic parameters of *Lc*ALT were studied, as described in the materials and methods. Interestingly, no hydrolytic activity was detected in the kinetic study: no free glucose was detected under glucose oxidase assay conditions. Similar observations were reported for *Lm*ALT, where very low hydrolytic activity (less than 5%) was noted^22^. *Lc*ALT exhibited Michaelis-Menten kinetics for transglycosylation, with sucrose as a substrate. The K_m_ and k_cat_ values for *Lc*ALT were 32.2 ± 3.2 mM and 290 ± 12 s^−1^, respectively. This K_m_ value for *Lc*ALT is approximately 10-fold higher than that of other glucansucrases, such as reuteransucrase^10^ and dextransucrase^30^, previously reported as 4.6 mM and 3 mM, respectively. Although approximate Michaelis–Menten kinetic parameters can be determined, the mechanism of glucansucrase is very complex and does not fully comply with Michaelis-Menten assumptions^31^.

### *Lc*-alternan product analysis

#### NMR analysis of linkage pattern in alternan produced by *Lc*ALT

Alternan produced by *Lc*ALT (*Lc*-alternan) was subjected to NMR analysis to determine the pattern of glycosidic linkages. *Lc*-alternan could not be dissolved in 1 M NaOD, unlike other insoluble glucans^32,33^. Nevertheless, at high concentrations some *Lc*-alternan could be dissolved in D_2_O for NMR analysis. Although it has high viscosity, which results in poor resolution of NMR signals, the viscosity of the *Lc*-alternan solution was reduced prior to NMR measurements using ultrasonication. To avoid the bias from differentially chemical hydrolysis of α-1,6 and α- 1,3 glycosidic bonds^34^.

**Figure 3:**
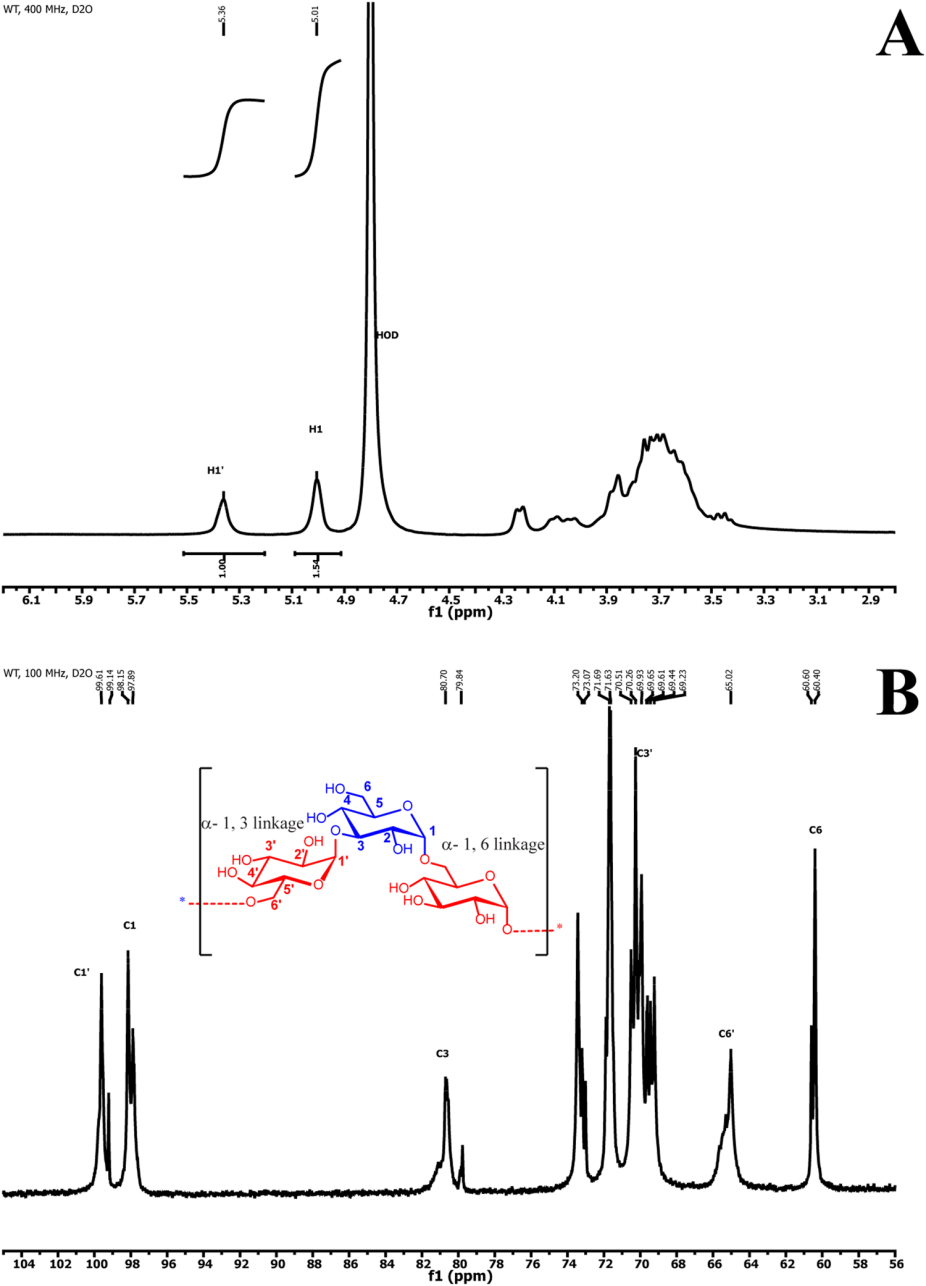
The NMR spectrum of *Lc*-alternan. (**A**) ^1^H NMR spectrum (400 MHz, D_2_O), and (**B**) ^13^C NMR spectrum (100 MHz, D_2_O).

Chemical shift ^13^C NMR spectrum at the anomeric region revealed at least four different types of anomeric carbons (Fig. 3B). Signals at δ 99.61 and 99.14 ppm correspond to anomeric carbon 1′ (C1′) of α-1,3 linkages, while signals at δ 98.15 and 97.89 ppm correspond to anomeric carbon 1 (C1) of the α-1,6 linkages^20^. These multiple signals with relatively high intensity of anomeric carbons at C1 and C1′ indicated the heterogeneous environments of linkages.

^1^H NMR spectra were acquired at 400 MHz (Fig. 3A and Supplemental Fig. S2). The anomeric protons at α-1,3 and α-1,6 linkages were identified as H1′ at δ 5.36 ppm and H1 at δ 5.01 ppm, respectively. The integration ratio of anomeric proton signals H1′: H1 in the *Lc-*alternan was 1:1.54. The integration ratio implied that there were 60% of H1 (α-1,6 linkage residue) and 40% of H1′ (α-1,3 linkage residue).

To further probe the chemical structure of *Lc*-alternan, ^1^H and COSY experiments were performed by using DMSO-d6 as solvent and a small amount of strong acid, trifluoroacetic acid (TFA). Gratifyingly, the TFA could accelerate proton exchange rate and distinguish the chemical shifts in the H1 anomeric proton region (δ 4.88–4.60 ppm)^35^. These peaks were clearly separated into four signals; δ 4.79, 4.76, 4.72, and 4.70 ppm. The COSY spectrum also revealed four strong coupling cross-peaks of four H2 protons (Supplemental Figs S3 and S4). Undoubtedly, this evidence reinforces our assumption that the *Lc*-alternan was distributed between α-1,3 and α-1,6-linked glucose residues in an irregular alternating manner.

Multiplicity-edited HSQC experiment (Supplemental Fig. S5) provided connectivity between protons and carbons on the carbohydrate skeleton. The multiplicity-edited HSQC informed not only the connectivity but also multiplicity of carbon nuclei, due to its nature of phase-sensitive experiment. In this measurement set-up, the presence of CH_2_ moieties appeared in blue cross-peaks while CH and CH_3_ moieties were in red cross-peaks. Thus, structural information of *Lc-*alternan was extracted and identified as C1′ (δ 99.58 ppm), C1 (δ 99.08 ppm), C3 (δ 80.39 ppm), C3′ (δ 70.47 ppm), C6′ (δ 65.21 ppm), and C6 (δ 60.49 ppm) for the carbon skeleton. Proton nucleus were elucidated as H1′ (δ 5.36 ppm), H1 (δ 5.01 ppm), H3′ (δ 4.22 ppm), H6′ (2 protons; δ 4.07, 3.69 ppm), H3 (δ 3.87 ppm), and H6 (δ 3.81 ppm). Additionally, HMBC spectrum (Supplemental Fig. S6) gave information of long-range connectivity between carbon and proton nuclei^36^. The H1′ signal at δ 5.36 ppm showed strong couplings with C3′ (δ 70.47 ppm) and inter-linkage coupling with C3 (δ 80.39 ppm). For another glucose residue, the H1 signal at δ 5.01 ppm also coupled with C3 (δ 80.39 ppm) and inter-linkage coupling was clear with C6′ (δ 65.21 ppm), thus it confirmed the previous assignments of the α-1,6 and α-1,3 linkages. However, several cross-peaks within anomeric proton regions of both H1′ (3 cross-peaks) and H1 (2 cross-peaks) were observed, which also implied the irregular structure of *Lc*-alternan.

### Methylation analysis and partial hydrolysis of *Lc*-alternan

**Figure 4:**
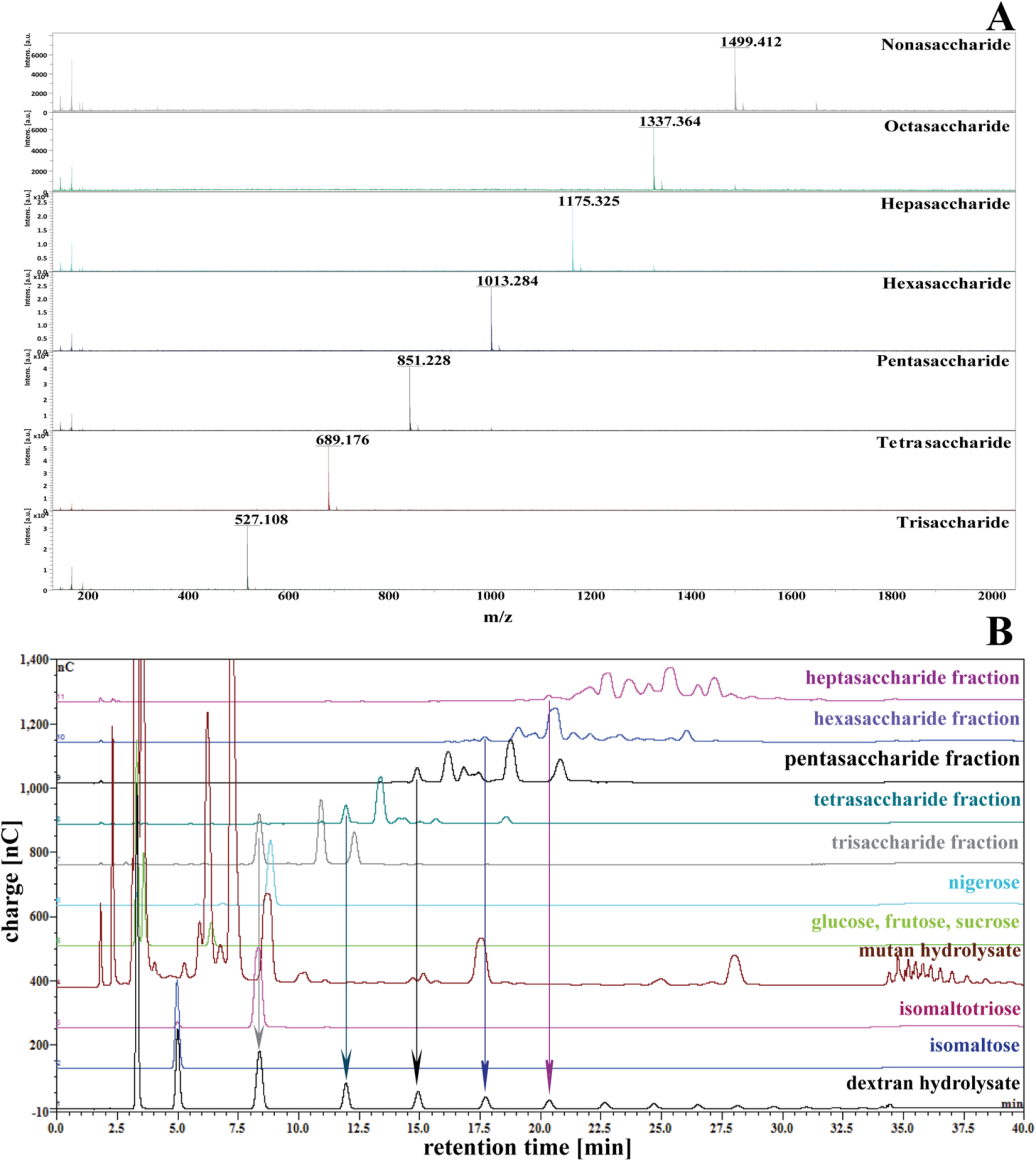
Analysis of partially hydrolysed *Lc*-alternan. (**A**) Mass analysis by MALDI-TOF and (**B**) product pattern analysis by HPAEC-PAD.

To ensure the complete methylation of *Lc*-alternan, the methylation reactions were performed twice prior to analysis. The results showed that *Lc*-alternan exhibited terminally substituted C3, C6 and C3,6 of methylated glucoses with different degrees as shown in Table 1. Obviously, the overall ratios of methylated glucose from *Lc*-alternan similar to that of *L*. *reuteri* strain 180 (EPS180)^37^ even though they shared only 50% identity of their amino acid sequences. Furthermore, sonication of *Lc*-alternan to increase its solubility prior to methylation analysis gave no significant methylation ratio of original to sonicated *Lc*-alternan, suggesting that methylation of the polymeric form was complete. However, several types of glucan comprise both α-1,6 and α-1,3 linkages with different ratios accounting for different linkage and branching patterns (Table 1). To further confirm that the glucose residues in *Lc*-alternan were not regularly linked by alternating α-1,6 and α-1,3 linkages, purified fractions of tri-, tetra-, penta-, hexa- and heptasaccharide from partially hydrolysed *Lc*-alternan were characterised by MALDI-TOF (Supplemental Figs S7, S8 and Fig. 4A), which showed the single oligomer size in each fraction. In addition, according to the high resolving power of HPAEC-PAD, products with different linkages could be separated. We found tri-, tetra-, penta-, hexa- and heptasaccharides in the product mixtures as indicated by the arrows in Fig. 4B. These results clearly indicated that dextran fragments up to heptasaccharide can be found in the *Lc*-alternan backbone. Moreover, this also suggested that structure of *Lc*-alternan is consistent with the proposed structure of glucan produced by *L*. *reuteri* strain 180^37^ or *L*. *brevis* E25^38^ rather than alternan product of *L*. *mesenteroides* NRRL-1355 (*Lm*-alternan)^18,20^. Nevertheless, structural analysis of acid-hydrolysed alternan is somewhat complicated because the acid itself possibly preferentially acts on some particular linkages^34^. Hence, some important oligosaccharide might have disappeared during this process.

**Table 1:** Mole percentage of methylated glucoses in hydrolysates of glucans.

Glucansucrase source	Glucopyranose methylation (%)						reference
	terminal	3-substituted	6-substituted	3,6-substituted	2-substituted	3,4-substituted	
*L*. *citreum* ABK-1	16	26	47	11	N/A	N/A	This work
*L*. *citreum* ABK-1 (sonicated polymer)	12	27	50	10	N/A	N/A	This work
*L*. *mesenteroides* NRRL-B1355	13	35	40	11	N/A	N/A	^38^
*L*. *reuteri* strain 180	12	24	52	12	N/A	N/A	^37^
*L*. *citreum* SK24.002	16	33	41	8	2	N/A	^47^
*L*. *mesenteroides* NRRL-B1118	15	44	29	9	N/A	4	^32^
*L*. *brevis* E25	19	19	54	8	N/A	N/A	^38^

### Solubility, nanoparticle assembly and film-forming ability of *Lc*-alternan

**Figure 5:**
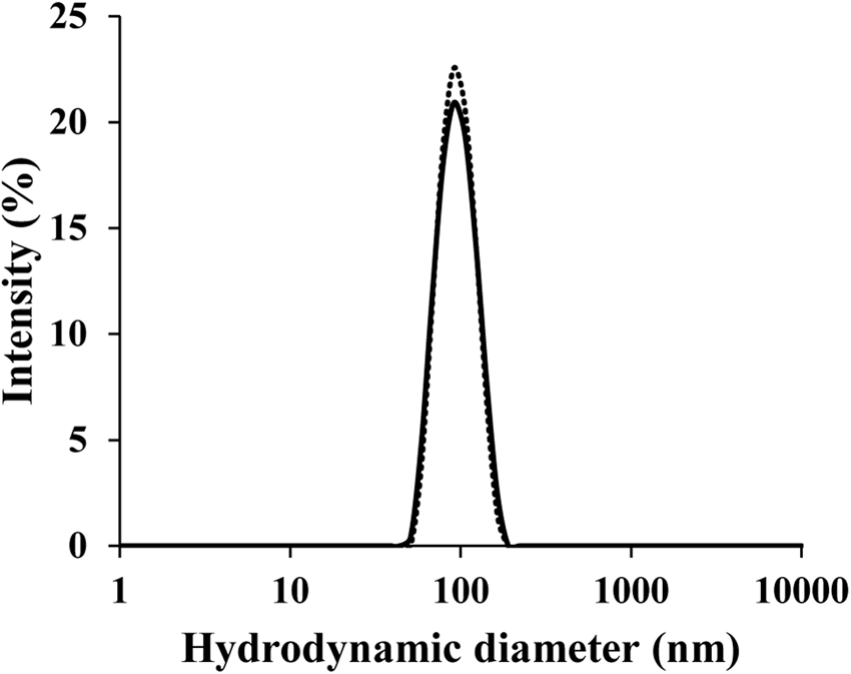
Particle size analysis of *Lc*-alternan nanoparticles by DLS. *Lc-*alternan was produced using 20% (w/v) sucrose incubated with 5 U of *Lc*ALT per gram of sucrose, for overnight at 37 °C. *Lc-*alternan was dialysed against deionised water then analysed by Zetasizer Nano ZS (Malvern) or lyophilised then re-dispersed into 1% (w/v) in deionised water prior to analysis. The particle size analysis of dialysed *Lc-*alternan nanoparticles was shown as a solid line (−), and *Lc-*alternan nanoparticles from the re-dispersed lyophilised form was shown as a dashed line (∙∙∙).

**Figure 6:**
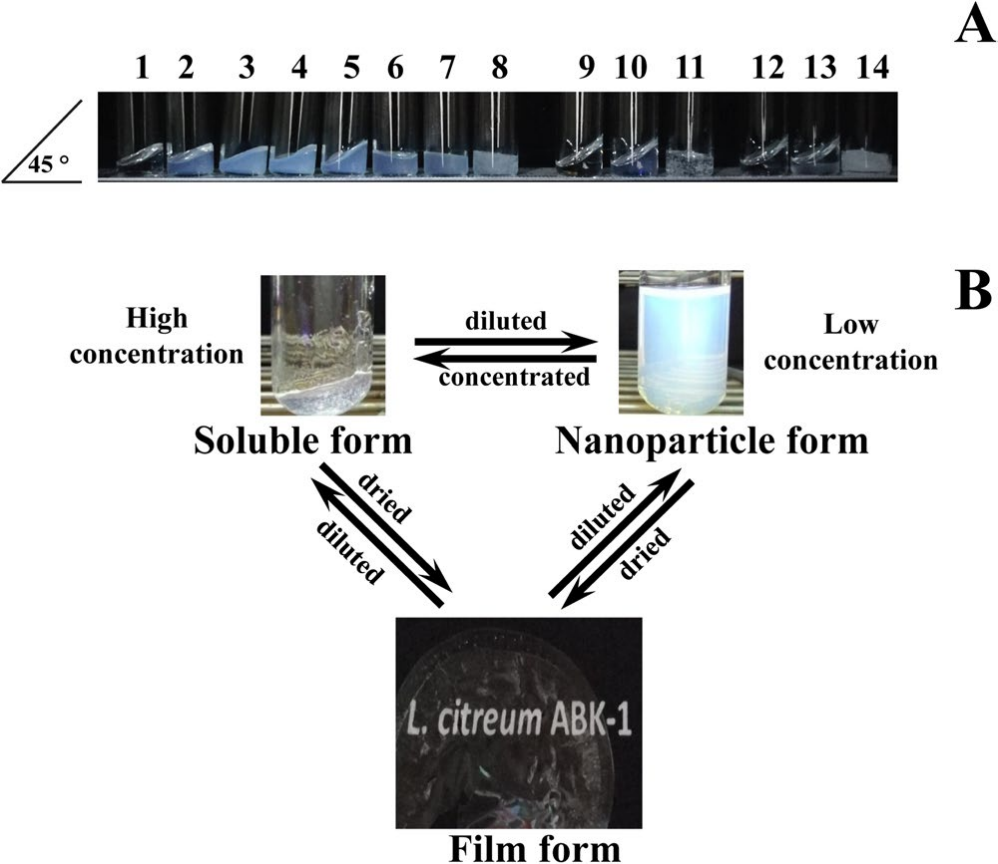
Solubility, nanoparticle and film formation of *Lc-*alternan. The effect of concentration and solvent on the solubility of *Lc-*alternan was performed, as described in materials and methods. (**A**) Tubes 1–8 present 0, 1, 2.5, 5, 10, 15, 20 and 25% (w/v) *Lc*-alternan in deionised water, tube 9–11 present 0, 1 and 20% (w/v) *Lc*-alternan in DMSO, and tube 12–14 show 0, 1 and 20% (w/v) *Lc*-alternan in 50% (w/v) NaOH, respectively. The tubes were at a 45-degree angle. (**B**) Interconversion of *Lc*-alternan among its three physical forms: aqueous solution, nanoparticles and film.

*Lc-*alternan at 1% (w/v) was insoluble in deionised water and DMSO, but it was partially soluble in 50% (w/v) NaOH. Furthermore, when *Lc-*alternan was re-suspended in aqueous solution, it goes through a self-assembly process, forming nanoparticles with an average size of 90 nm, at low concentrations (less than 10% (w/v)) as shown in Fig. 5, and did not precipitate as insoluble glucan (such as mutan polymer from *S*. *sorbinus*^28^, Supplemental Fig. S9), which consists of mainly α-1,3 linkage (Supplemental Fig. S10). When the concentration of *Lc-*alternan was increased, it became more soluble, increasing the viscosity of the solution. An opaque gel was observed at concentrations above 15% (w/v), as shown in Fig. 6A. Nanoparticle assembly and increasing solubility at high concentrations have also been observed in alternan produced from *L*. *mesenteroids* NRRL-1335^39^ and *L*. *citreum* SK24.002^40^.

Surprisingly, conversion between nanoparticle and gel formation of *Lc-*alternan was reversible in a concentration-dependent manner. This evidence is not consistent with other well-known carbohydrate polymers such as starch and dextran, etc. Moreover, these molecules are freely dissolved at low concentration. Furthermore, *Lc-*alternan nanoparticles form a clear transparent film upon drying although the film itself is rather fragile. This *Lc*-alternan film can be readily solubilised, forming a clear solution at high concentration and re-assemble into nanoparticles when further diluted to low concentration (less than 10% (w/v)) (Fig. 6B). Interestingly, if the concentration of *Lc-*alternan was increased to 20% (w/v) in DMSO, *Lc-*alternan formed a clear solution with high viscosity. Nonetheless, the *Lc*-alternan nanoparticles cannot be observed under transmission electron microscope (TEM) since the *Lc*-alternan nanoparticles is only present in solution phase not in solid phase provided by this technique (Supplemental Fig. S11).

Though other polymers such as dextran^41,42^, chitosan^43^ and cellulose^44^ have been reported to be able to form nanoparticles and have been applied as encapsulation agents with success, additional processes such as chemical modification or solvent exchange were needed. The properties of *Lc-*alternan and its ability to shift into different forms, simply by changing its concentration make *Lc-*alternan a very promising candidate for encapsulation applications or concentration-dependent sensor materials.

The linear polymers with various sizes were purified by GPC. The masses detected by MALDI-TOF corresponded to the sizes of linear structure of permethylated polymers (Supplemental Fig. S12A). Interestingly, when unmodified polymers were analysed by MALDI-TOF, the acquired m/z corresponded to cyclic structures were observed (Supplemental Fig. S12B). These suggested that the cyclization is an artefact of MALDI-TOF ionisation (probably catalysed by the presence of dihydroxybenzoic acid (DHB) used as a matrix).

The nanoparticle and solubility properties of *Lc-*alternan in water may arise from the overall structure of its polymer backbone, promoting strong intra-molecular interactions and packaging of the polymer chains. These interactions could be a result of the insertion of α-1,3 glycosidic linkages into backbone, as dextran containing mainly α-1,6 linkages is known to be freely soluble in aqueous solution^19^. Furthermore, glucans containing high ratio of α-1,3 linkage were reported to be insoluble, such as glucans from *L*. *mesenteroides* NRRL B-1355^11^, *S*. *mutans*^45^, *S*. *sorbinus*^28,46^ and *L*. *mesenteroides* NRRL B-1118^32^. Interestingly, the nanoparticle formation and solubility properties of *Lc*-alternan appear to be dependent on the chain length, since sonicated *Lc*-alternan with shorter chain length^39^ no longer forms nanoparticle in solution.

## Conclusion

The full length alternansucrase gene from *Leuconostoc citreum* ABK-1 was successfully cloned, expressed in *E*. *coli* and *Lc*ALT was biochemically characterised. This report provides an example of glucansucrase diversity in lactic acid bacteria as well as the unusual relationship between carbohydrate structures and their properties. The properties of *Lc*-alternan makes it a promising carbohydrate polymer, which may be applied for nanotechnology, encapsulation and control released technology, as well as the food and pharmaceutical industries.

## Electronic supplementary material

supplementary data
