# Supplementary material for: An α-1,6-and α-1,3-linked glucan produced by Leuconostoc citreum ABK-1 alternansucrase with nanoparticle and film-forming properties
Source: Sci Rep. 2018 May 29;8:8340. doi: 10.1038/s41598-018-26721-w (PMC5974361; doi:10.1038/s41598-018-26721-w)
Supplement: Supplementary file 1 — supplementary data [file 41598_2018_26721_MOESM1_ESM.pdf]

Supplementary data

An α-1,6-and α-1,3-linked glucan produced by *Leuconostoc citreum* ABK-1 alternansucrase with nanoparticle and film-forming properties

Karan Wangpaiboon[1], Panuwat Padungros[2], Santhana Nakapong[3], Thanapon Charoenwongpaiboon[1], Martin Rejzek[4], Robert A. Field[4] and Rath Pichyangkura[1,*]

[1]*Department of Biochemistry, Faculty of Science, Chulalongkorn University, Bangkok 10330, Thailand*

[2]*Department of Chemistry, Faculty of Science, Chulalongkorn University, Bangkok 10330, Thailand*

[3]*Department of Chemistry, Science, Ramkhamhaeng University, Bangkok 10240, Thailand*

[4]*Department of Biological Chemistry, John Innes Centre, Norwich Research Park, Norwich NR4 7UH, UK*

**Corresponding author: prath@chula.ac.th*


```
LcALT    MEQQETVTRKKLYKSGKVWVAAATAFAVLGVSTVTTVNADTNPNVAVKQINNTGTNDSGE
LmALT    MKQQETVTRKKLYKSGKVWVAAATAFAVLGVSTVTTVHADTNSNVAVKQINNTGTNDSGE
         *:***********************************:**** *****************

LcALT    KKAPVPSTNNDSLKQGTDGFWYDSDGNRVDQKTNQILLTAEQLKKNNEKNLSVISDDTSK
LmALT    KKVPVPSTNNDSLKQGTDGFWYDSDGNRVDQKTNQILLTAEQLKKNNEKNLSVISDDTSK
         **.*********************************************************

LcALT    KDDENISKQTKIANQQTVDTAKGLTTSNLSDPITGGHYENHNGYFVYIDASGKQVTGLQN
LmALT    KDDENISKQTKIANQQTVDTAKGLTTSNLSDPITGGHYENHNGYFVYIDASGKQVTGLQN
         ************************************************************

LcALT    IDGNLQYFDDNGYQVKGSFRDVNGKHIYFDSVTGKASSNVDIVNGKAQGYDAQGNQLKKS
LmALT    IDGNLQYFDDNGYQVKGSFRDVNGKHIYFDSVTGKASSNVDIVNGKAQGYDAQGNQLKKS
         ************************************************************

LcALT    YVADSSGQTYYFDGNGQPLIGLQTIDGNLQYFNQQGVQIKGGFQDVNNKRIYFAPNTGNA
LmALT    YVADSSGQTYYFDGNGQPLIGLQTIDGNLQYFNQQGVQIKGGFQDVNNKRIYFAPNTGNA
         ************************************************************

LcALT    VANTEIINGKLQGRDANGNQVKNAFSTDVAGNTFYFDANGVMLTGLQTISGKTYYLDEQG
LmALT    VANTEIINGKLQGRDANGNQVKNAFSKDVAGNTFYFDANGVMLTGLQTISGKTYYLDEQG
         **************************.*********************************
```

```
LcALT    HLRKNYAGTFNNQFMYFDADTGAGKTAIEYQFDQGLVSQSNENTPHNAAKSYDKSSFENV
LmALT    HLRKNYAGTFNNQFMYFDADTGAGKTAIEYQFDQGLVSQSNENTPHNAAKSYDKSSFENV
         ************************************************************

LcALT    DGYLTADTWYRPTDILKNGDTWTASTETDMRPLLMTWWPDKQTQANYLNFMSSKGLGITT
LmALT    DGYLTADTWYRPTDILKNGDTWTASTETDMRPLLMTWWPDKQTQANYLNFMSSKGLGITT
         ************************************************************

LcALT    TYTAATSQKTLNDAAFVIQTAIEQQISLKKSTEWLRDAIDSFVTTQANWNKQTEDEAFDG
LmALT    TYTAATSQKTLNDAAFVIQTAIEQQISLKKSTEWLRDAIDSFVKTQANWNKQTEDEAFDG
         *******************************************.****************

LcALT    LQWLQGGFLAYQDDSHRTPNTDSGNNRKLGRQPVNIDGSKDTTDGKGSEFLLANDIDNSN
LmALT    LQWLQGGFLAYQDDSHRTPNTDSGNNRKLGRQPINIDGSKDTTDGKGSEFLLANDIDNSN
         *********************************:**************************

                                             ★
LcALT    PIVQAEQLNWLHYLMNFGSITGNNDNANFDGIRVDAVDNVDADLLKIAGDYFKALYGTDK
LmALT    PIVQAEQLNWLHYLMNFGSITGNNDNANFDGIRVDAVDNVDADLLKIAGDYFKALYGTDK
         ************************************************************

                      ★
LcALT    SDANANKHLSILEDWNGKDPQYVNQQGNAQLTMDYTVTSQFGNSLTHGANNRSNMWYFLD
LmALT    SDANANKHLSILEDWNGKDPQYVNQQGNAQLTMDYTVTSQFGNSLTHGANNRSNMWYFLD
         ************************************************************

                                                       ★
LcALT    TGYYLNGDINKKIVDKNRQNSGTLVNRIANAGDTQVIPNYSFIRAHDYDAQDPIRRAMID
LmALT    TGYYLNGDLNKKIVDKNRPNSGTLVNRIANSGDTKVIPNYSFVRAHDYDAQDPIRKAMID
         ********:********* ***********:***:*******:************:****

LcALT    HGIIKNMQDTFTFDQLAQGMEFYYQDQNNPSGFKKYNDYNLPSAYAMLLTNKDTIPRVYY
LmALT    HGIIKNMQDTFTFDQLAQGMEFYYKDQENPSGFKKYNDYNLPSAYAMLLTNKDTVPRVYY
         ************************:**:**************************:*****

LcALT    GDMYYEGGQYMQNETIYNRVISALLKARIKYVSGGQTMATDSSGKDLKDGETDLLTSVRF
LmALT    GDMYLEGGQYMEKGTIYNPVISALLKARIKYVSGGQTMATDSSGKDLKDGETDLLTSVRF
         **** ******:: **** *****************************************

LcALT    GKGIMTSDQTTTQDNSQDYKNQGIGVIVGNNPDLKLNNDKTITLHMGKAHKNQLYRALAL
LmALT    GKGIMTSDQTTTQDNSQDYKNQGIGVIVGNNPDLKLNNDKTITLHMGKAHKNQLYRALVL
         **********************************************************.*

LcALT    SNDSGIDVYNSDDEAPTLRTNDNGDLIFHKTNTFVKQDGTIINYEMKGSLNALISGYLGV
```

```
LmALT      SNDSGIDVYDSDDKAPTLRTNDNGDLIFHKTNTFVKQDGTIINYEMKGSLNALISGYLGV
           *********:***:**********************************************

LcALT      WVPVGASDSQDARTVATEASSSNDGSVFHSNAALDSNVIYEGFSNFQAMPTSPEQSTNVV
LmALT      WVPVGASDSQDARTVATESSSSNDGSVFHSNAALDSNVIYEGFSNFQAMPTSPEQSTNVV
           ******************:*****************************************

LcALT      IAANAEMFKKLGITSFELAPQYRSSGDTNYGGMSFLDSFLNNGYAFTDRYDLGFNKADGT
LmALT      IATKANLFKELGITSFELAPQYRSSGDTNYGGMSFLDSFLNNGYAFTDRYDLGFNKADGN
           **::*::**:*************************************************.

LcALT      PNPTKYGTDQDLRNAIEALHKNGMQAIADWVPDQIYALPGKEVVTATRVDERGNQLKDTD
LmALT      PNPTKYGTDQDLRNAIEALHKNGMQAIADWVPDQIYALPGKEVVTATRVDERGNQLKDTD
           ************************************************************

LcALT      FVNLLYVANTKSSGVDYQSKYGGEFLDKLKEEYSSLFKQNQVSTGQPIDASTKIKQWSAK
LmALT      FVNLLYVANTKSSGVDYQAKYGGEFLDKLREEYPSLFKQNQVSTGQPIDASTKIKQWSAK
           ******************:**********:*** **************************

LcALT      YMNGTNILHRGAYYVLKDWATNQYFNVAKTDEVFLPLQLQNKDPQTGFISDASGVKYYSI
LmALT      YMNGTNILHRGAYYVLKDWATNQYFNIAKTNEVFLPLQLQNKDAQTGFISDASGVKYYSI
           **************************:***:************ ****************

LcALT      SGYQAKDTFIEDGNGNWYYFDKDGYMARSQQGENPIRTVETSVNTRNGNYYFMPNGVELR
LmALT      SGYQAKDTFIEDGNGNWYYFDKDGYMVRSQQGENPIRTVETSVNTRNGNYYFMPNGVELR
           **************************.*********************************

LcALT      KGFGTDNSGNVYYFDDQGKMVRDKYINDDANNFYHLNVDGTMSRGLFKFDSDTLQYFASN
LmALT      KGFGTDNSGNVYYFDDQGKMVRDKYINDDANNFYHLNVDGTMSRGLFKFDSDTLQYFASN
           ************************************************************

LcALT      GVQIKDSYAKDSKGNKYYFDSATGNNDTVKAQAWDGNGYYITIDSDANNTIGVNTDYTAY
LmALT      GVQIKDSYAKDSKGNKYYFDSATGNNDTGKAQTWDGNGYYITIDSDANNTIGVNTDYTAY
           **************************** ***:***************************

LcALT      ITSSLREDGLFANAPYGVVTKDQNGNDLKWQYINHTKQYEGQQVQVTRQYTDSKGVSWNL
LmALT      ITSSLREDGLFANAPYGVVTKDQNGNDLKWQYINHTKQYEGQQVQVTRQYTDSKGVSWNL
           ************************************************************

LcALT      ITFAGGDLQGQKLWVDSRALTMTPFKTMNQISFISYANRNDGLFLNAPYQVKGYQLAGMS
LmALT      ITFAGGDLQGQRLWVDSRALTMTPFKTMNQISFISYANRNDGLFLNAPYQVKGYQLAGMS
           ***********:************************************************
```

```
LcALT       NQYKGQQVTIAGVANVSGKDWSLISFNGTQYWIDSQALNTNFTHDMNQKVFVNTTSNLDG
LmALT       NQYKGQQVTIAGVANVSGKDWSLISFNGTQYWIDSQALNTNFTHDMNQKVFVNTTSNLDG
            ************************************************************

LcALT       LFLNAPYRQPGYKLAGLAKNYNNQTVTVSQQYFDDQGTVWSQVVLGGQTVWVDNHALAQM
LmALT       LFLNAPYRQPGYKLAGLAKNYNNQTVTVSQQYFDDQGTVWSQVVLGGQTVWVDNHALAQM
            ************************************************************

LcALT       QVSDTSQQLYVNSNGRNDGLFLNAPYRGQGSQLIGMTADYNGQHVQVTKQGQDAYGAQWR
LmALT       QVSDTDQQLYVNSNGRNDGLFLNAPYRGQGSQLIGMTADYNGQHVQVTKQGQDAYGAQWR
            *****.******************************************************

LcALT       LITLNNQQVWVDSRALSTTIMQAMNDDMYVNSNQRTDGLWLNAPYTMSGAKWAGDTRSAN
LmALT       LITLNNQQVWVDSRALSTTIMQAMNDNMYVNSSQRTDGLWLNAPYTMSGAKWAGDTRSAN
            **************************:*****.***************************


LcALT       GRYVHISKAYSNEVGNTYYLTNLNGQSTWIDKRAFTATFDQVVALNATIVARQRPDGMFK
LmALT       GRYVHISKAYSNEVGNTYYLTNLNGQSTWIDKRAFTVTFDQVVALNATIVARQRPDGMFK
            ************************************.***********************

LcALT       TAPYGEAGAQFVDYVTNYNQQTVPVTKQHSDAQGNQWYLATVNGTQYWIDQRSFSPVVTK
LmALT       TAPYGEAGAQFVDYVTNYNQQTVPVTKQHSDAQGNQWYLATVNGTQYWIDQRSFSPVVTK
            ************************************************************

LcALT       VVDYQAKIVPRTTRDGVFSGAPYGEVNAKLVNMATAYQNQVVHATGEYTNASGITWSQFA
LmALT       VVDYQAKIVPRTTRDGVFSGAPYGEVNAKLVNMATAYQNQVVHATGEYTNASGITWSQFA
            ************************************************************

LcALT       LSGQEDKLWIDKRALQA
LmALT       LSGQEDKLWIDKRALQA
            *****************
```

Fig. S1 Pairwise alignment between *Lc*ALT and *Lm*ALT amino acid sequences. The 2,057 residues of *Lc*ALT and *Lm*ALT were aligned using Clustal OMEGA. Catalytic domain sequences of GH70 were shown in red and different residues between *Lc*ALT and *Lm*ALT were highlighted in yellow. Three conserved motifs were underlined and three catalytic residues were marked by stars.

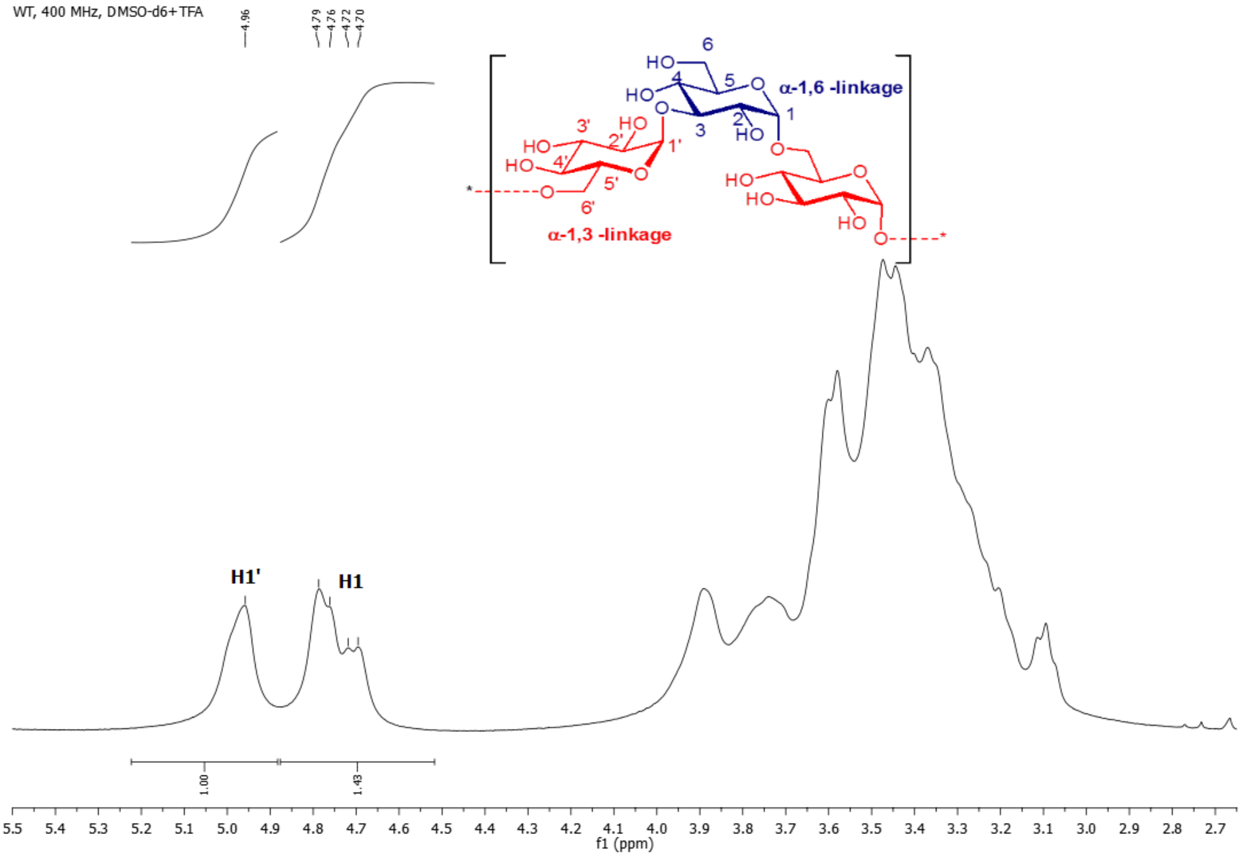

Fig. S2 ^{1}H NMR spectrum of *Lc*- alternan (400 MHz, DMSO-d6+TFA)

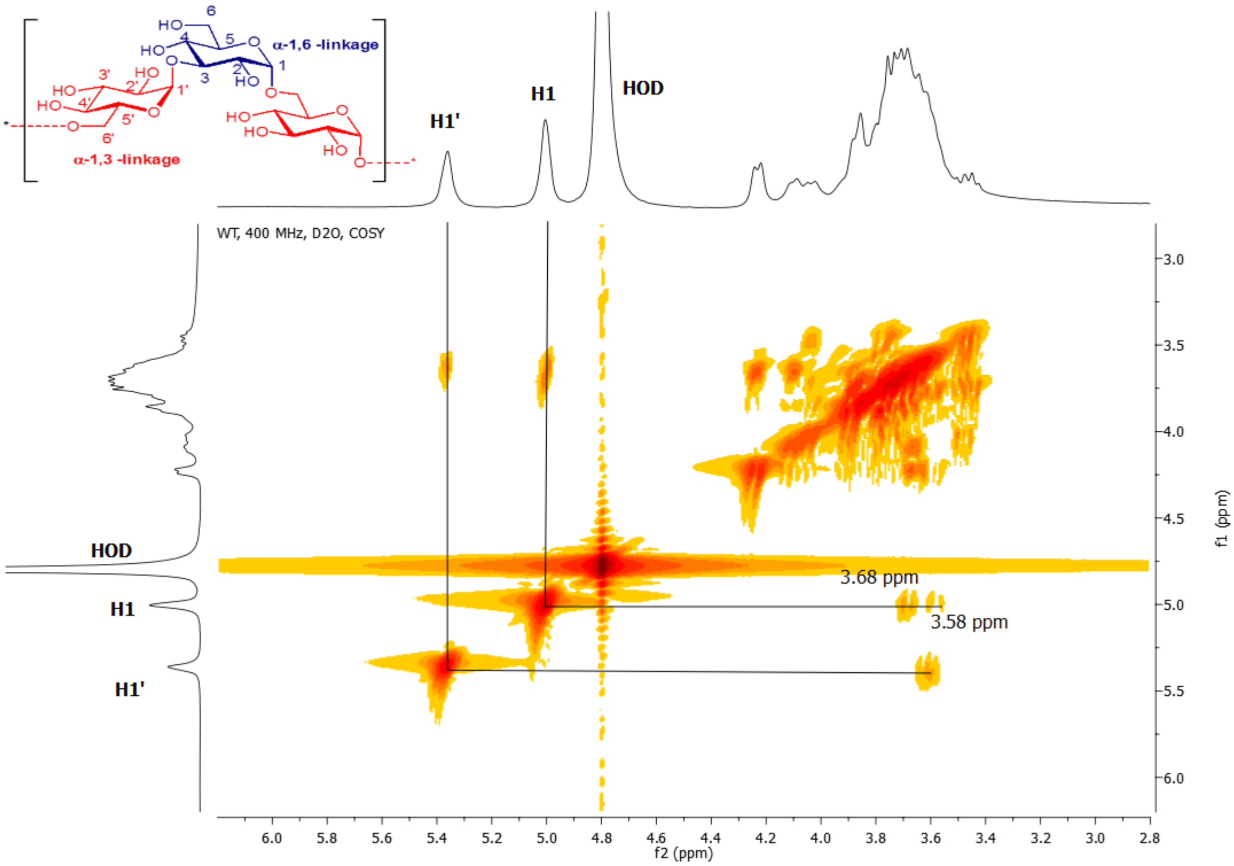

Fig. S3 COSY spectrum of *Lc*- alternan (400 MHz, D_2O)

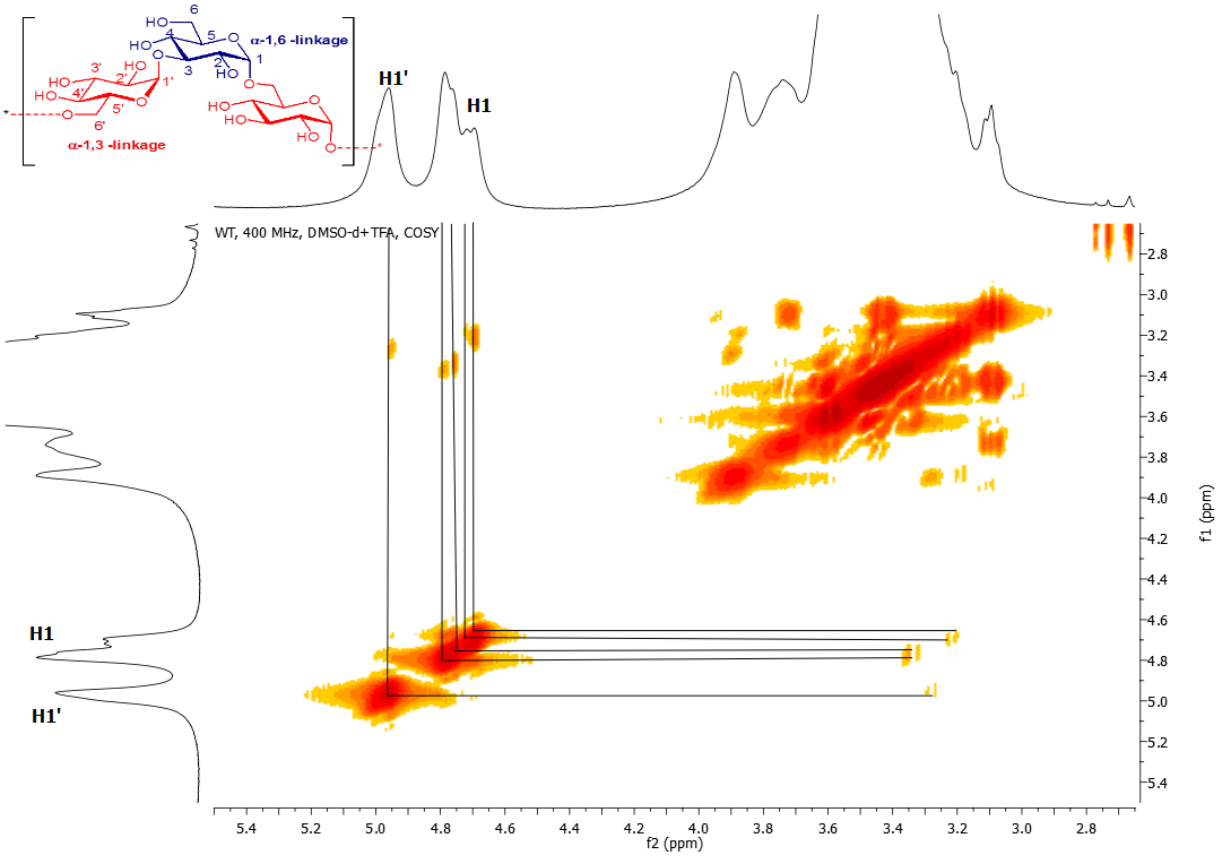

Fig. S4 COSY spectrum of *Lc*- alternan (400 MHz, DMSO-d6+TFA)

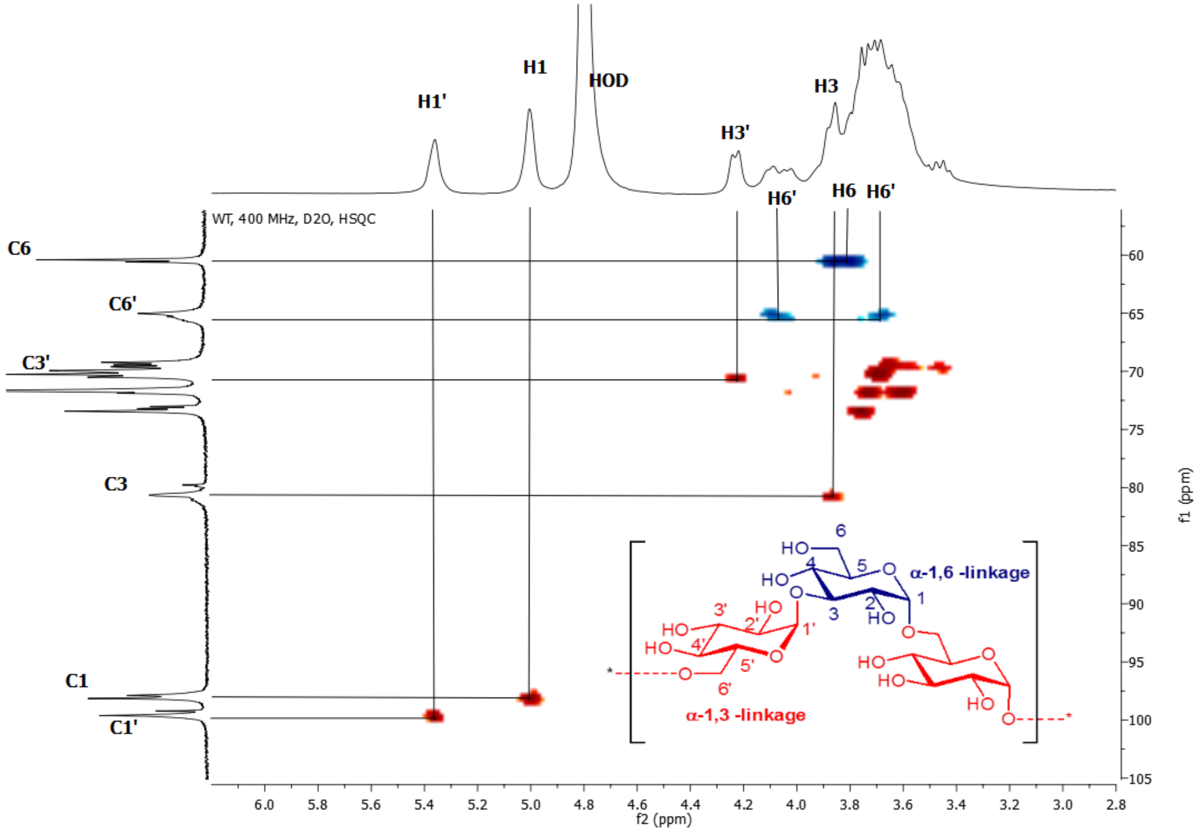

Figure S5. Multiplicity-edited HSQC spectrum of *Lc*- alternan (400 MHz, D_2O)

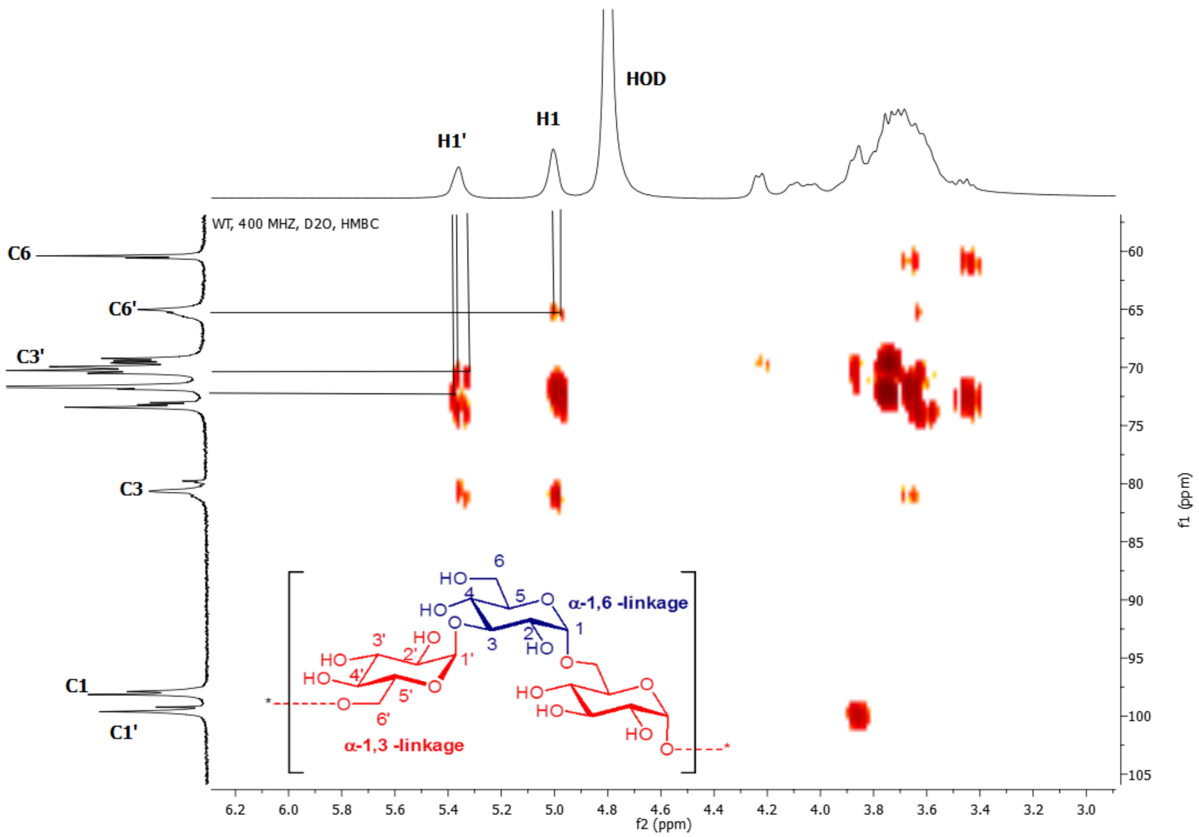

Figure S6. HMBC spectrum of *Lc*- alternan (400 MHz, D_2O)

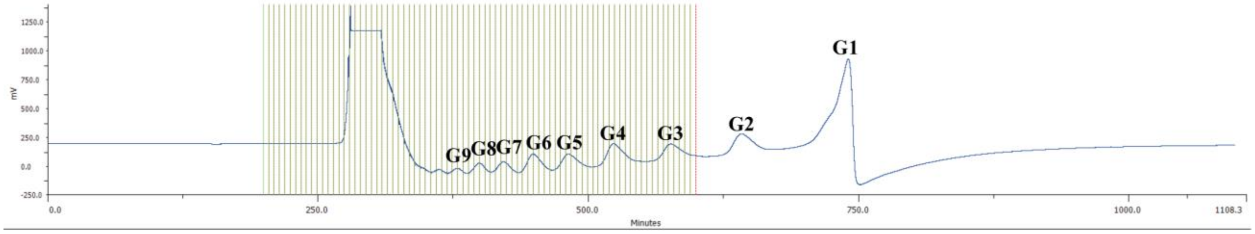

Figure S7. Separation of partially hydrolysed products by size exclusion chromatography. The partially hydrolysed alternan polymers were separated using Biogel P-2 column at 50 °C with flow rate of 0.5 mL/min. Blue line represents signal from RI detector. The 2.5-ml fraction size was collected between 200 – 600 min.

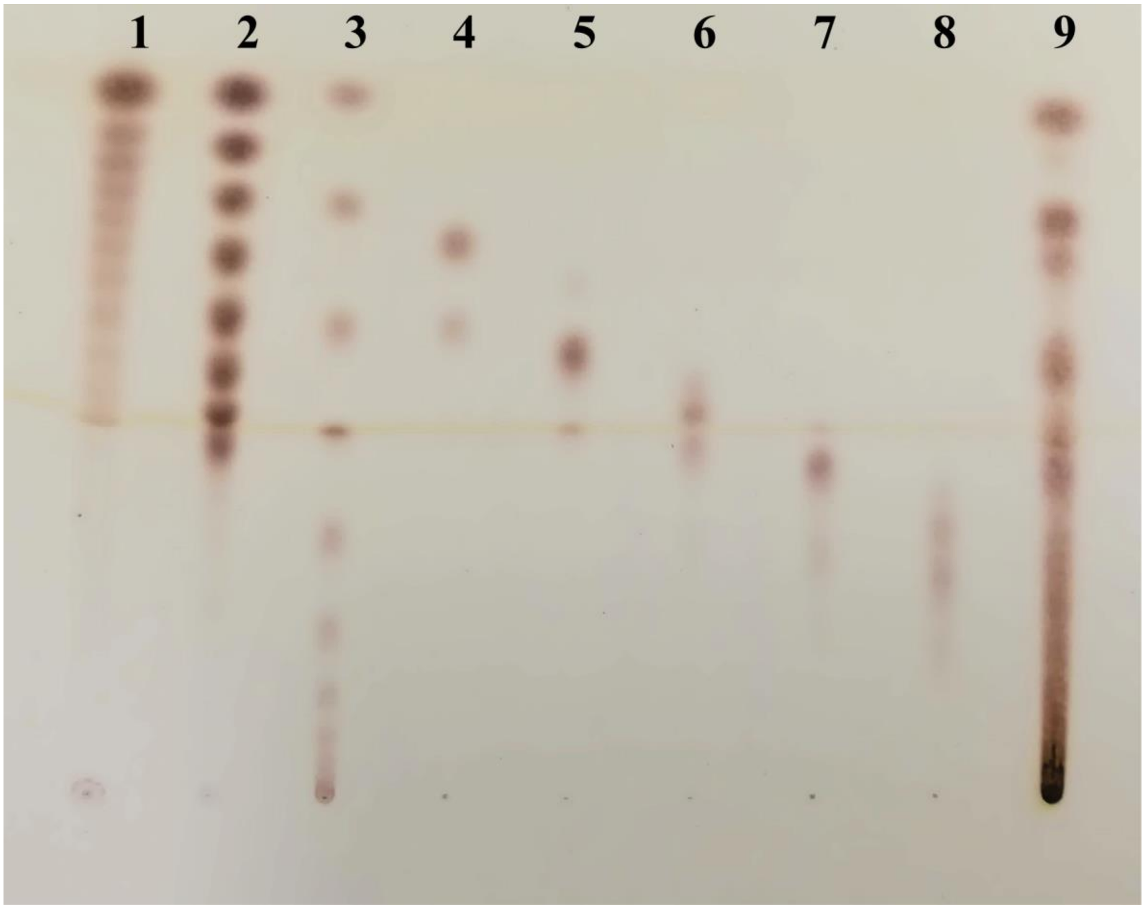

Figure S8. TLC analysis of oligo *Lc*-alternan separated by Biogel P-2 column. Lane: 1, 2 and 3 are partial hydrolysis of mutan polymer from *S. sobrinus* MFe28 (obtained from acetolysis), std. maltooligosaccharide (G1 - G8) and partial hydrolysis of dextran polymer from *Leuconostoc* spp. (Sigma), respectively. Lane: 4 – 8 are products from fraction No. 77, 66, 58, 52 and 46 (G3 – G7, respectively), and lane 9 is partially hydrolysed *Lc*-alternan. The TLC was run for 2 ascent in mobile phase, acetonitrile:ethylacetate:1-propanol:water (85:20:50:60), and then developed by orcinol solution, (2 % (w/v) orcinol in H_2SO_4:Ethanol:water (11:84:5.5)).

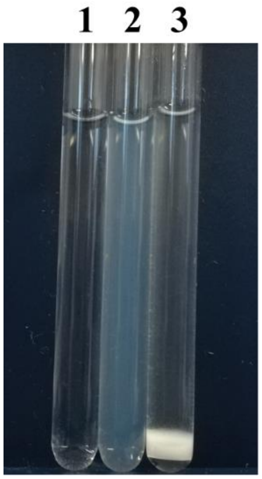

Figure S9. Comparison of solubility of three glucan polymers in water. Tube 1: 1% (w/v) of dextran polymer from *Leuconostoc* spp. (Sigma), tube 2: 1% (w/v) of *Lc*-alternan, and tube 3: 1% (w/v) of insoluble glucan from *S. sobrinus*, dissolved or suspended in water and stand still for 15 min.

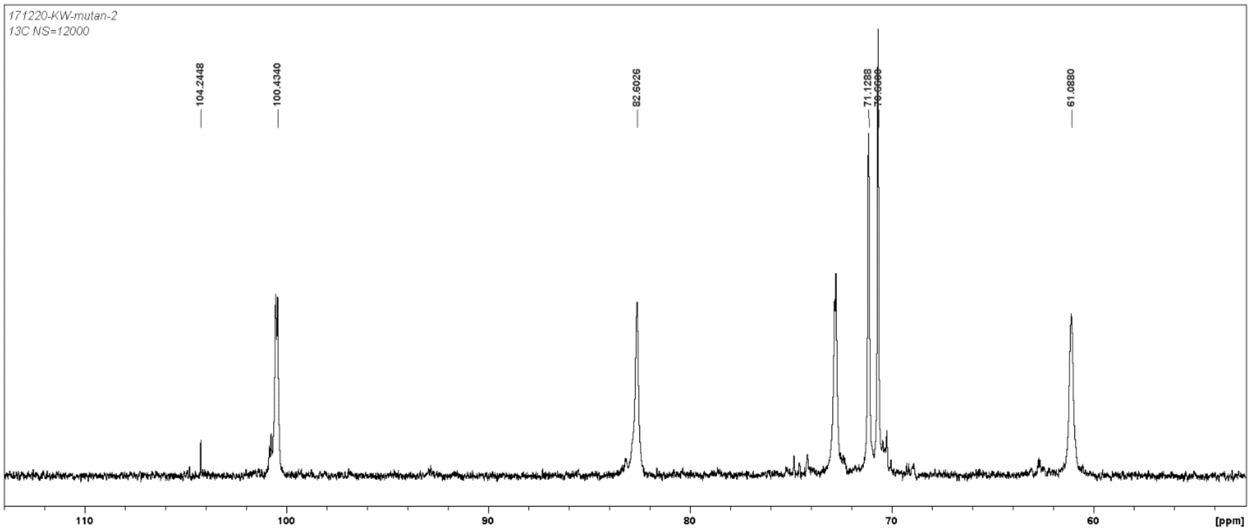

Figure S10. ^{13}C NMR spectrum of insoluble glucan (mutan polymer) from *S. sobrinus* (100 MHz, 1 M NaOD). The polymer was prepared from 10 % of sucrose in 50 mM acetate buffer pH 5.5 using 2 U of enzyme per gram of sucrose, incubated at 37 °C for 16 hr. The polymer pellet was collected by centrifuging at 3000xg for 5 min. The pellet was suspended in deionised water and centrifuged for 5 min (repeat washing step for 5 times). Then, the polymer were lyophilised.

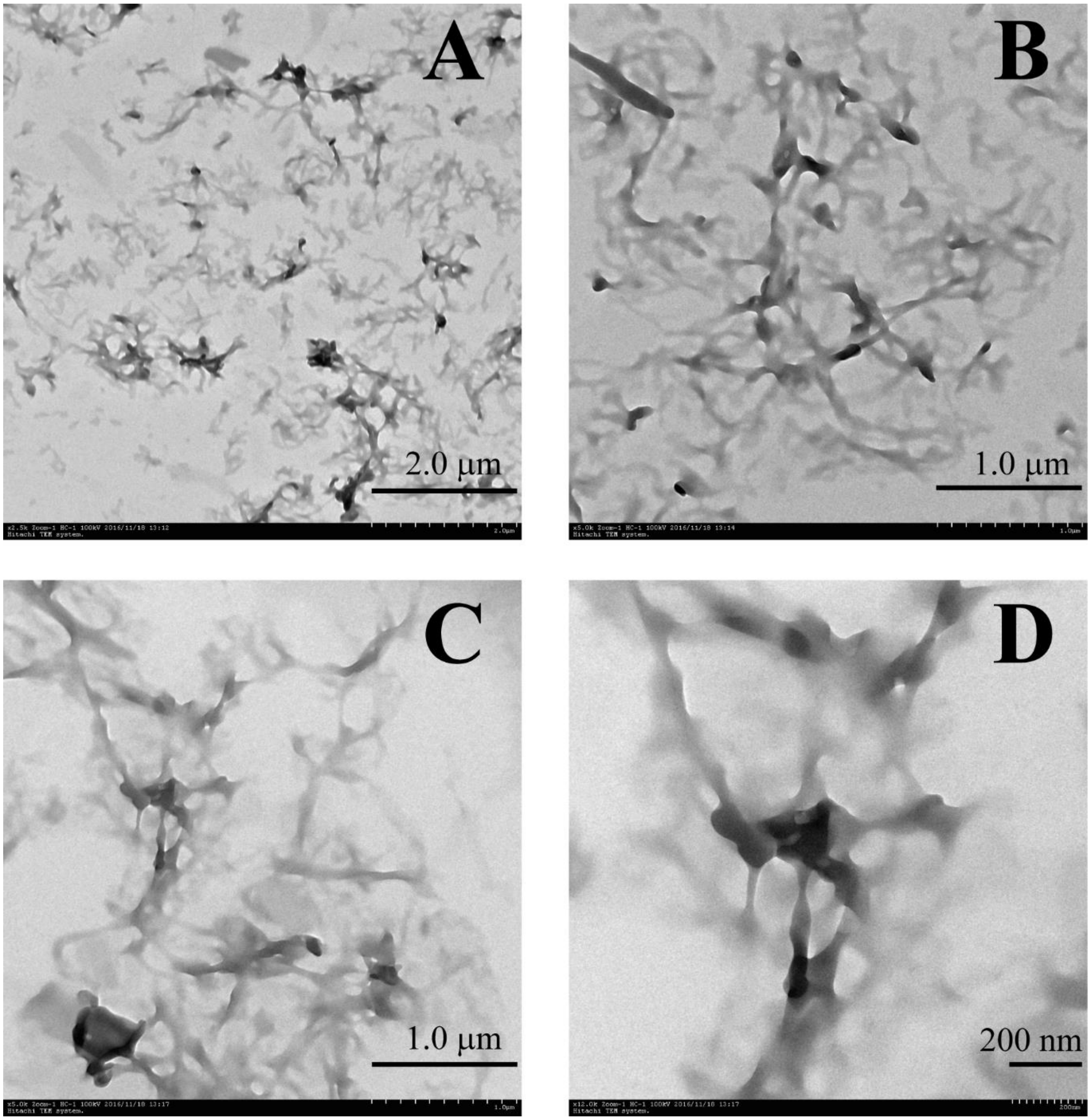

Figure S11. Images of *Lc*-alternan under transmission electron microscope (TEM). The *Lc*-alternan was observed with different magnifications; the panel (A) : 2.5 k, (B) and (C) : x 5.0 k and (D) : x 12.0 k magnification, respectively.

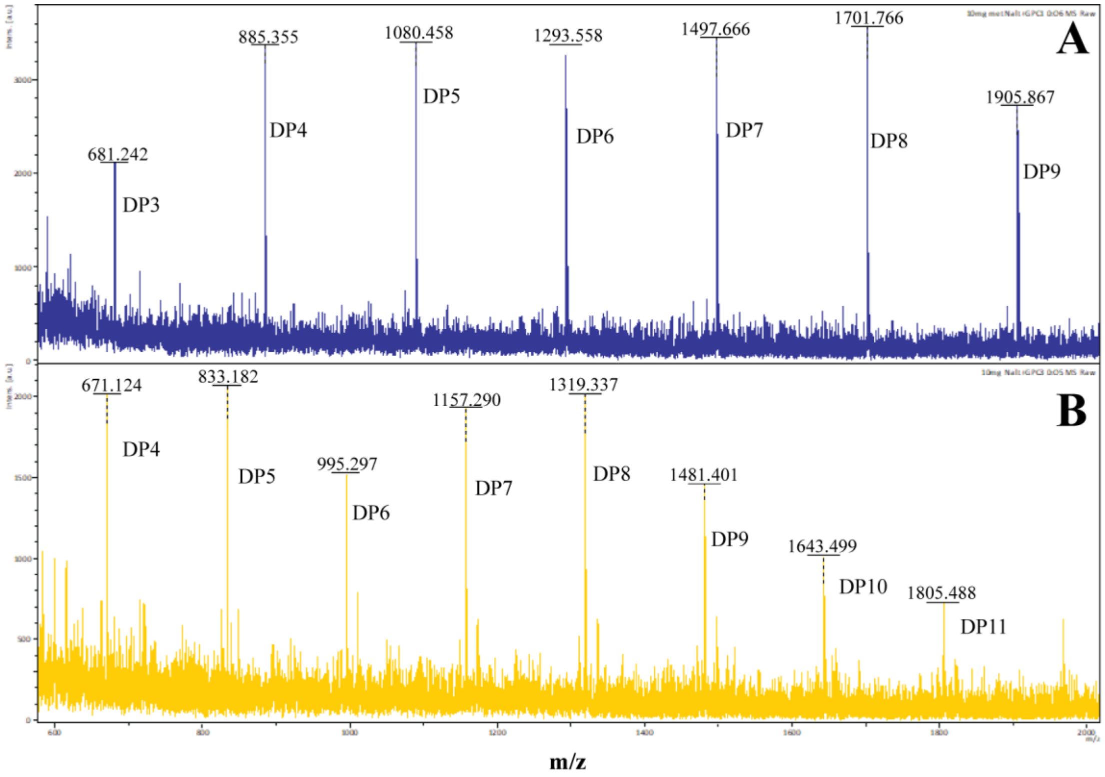

Figure S12. (A) MALDI-TOF of pre-methylated polymer and (B) MALDI-TOF of polymer without methylation. The dihydroxybenzoic aid (DHB) was used as a matrix.
